# Wearable accelerometers for measuring and monitoring the motor behaviour of infants with brain damage during CareToy-Revised training

**DOI:** 10.1186/s12984-023-01182-z

**Published:** 2023-05-06

**Authors:** Mattia Franchi de’ Cavalieri, Silvia Filogna, Giada Martini, Elena Beani, Martina Maselli, Matteo Cianchetti, Nevio Dubbini, Giovanni Cioni, Giuseppina Sgandurra, The CareToy-R Consortium, Claudia Artese, Veronica Barzacchi, Alessandra Cecchi, Marta Cervo, Maria Luce Cioni, Carlo Dani, Paolo Dario, Marco Di Galante, Ugo Faraguna, Patrizio Fiorini, Viola Fortini, Matteo Giampietri, Simona Giustini, Clara Lunardi, Irene Mannari, Valentina Menici, Letizia Padrini, Filomena Paternoster, Riccardo Rizzi

**Affiliations:** 1 Department of Developmental Neuroscience, IRCCS Fondazione Stella Maris, Viale del Tirreno 331, Calambrone, 56128 Pisa, Italy; 2 Tuscan Ph.D. Programme of Neuroscience, University of Florence, Florence, Italy; 3 The BioRobotics Institute, Scuola Superiore Sant’Anna, Pisa, Italy; 4 Department of Excellence in Robotics and AI, Scuola Superiore Sant’Anna, Pisa, Italy; 5 Miningful Studio s.r.l.s., Pisa, Italy; 6 Department of Clinical and Experimental Medicine, University of Pisa, Pisa, Italy

**Keywords:** Wearable sensors for healthcare, Accelerometers, Infant’s activity, Upper limb movements, Early detection, Early intervention, Cerebral palsy, CareToy system, Tele-rehabilitation, Functional data analysis

## Abstract

**Background:**

Nowadays, wearable sensors are widely used to quantify physical and motor activity during daily life, and they also represent innovative solutions for healthcare. In the clinical framework, the assessment of motor behaviour is entrusted to clinical scales, but they are dependent on operator experience. Thanks to their intrinsic objectivity, sensor data are extremely useful to provide support to clinicians. Moreover, wearable sensors are user-friendly and compliant to be used in an ecological environment (i.e., at home). This paper aims to propose an innovative approach useful to predict clinical assessment scores of infants’ motor activity.

**Materials and methods:**

Starting from data acquired by accelerometers placed on infants’ wrists and trunk during playtime, we exploit the method of functional data analysis to implement new models combining quantitative data and clinical scales. In particular, acceleration data, transformed into activity indexes and combined with baseline clinical data, represent the input dataset for functional linear models.

**Conclusions:**

Despite the small number of data samples available, results show correlation between clinical outcome and quantitative predictors, indicating that functional linear models could be able to predict the clinical evaluation. Future works will focus on a more refined and robust application of the proposed method, based on the acquisition of more data for validating the presented models.

*Trial registration number*: ClincalTrials.gov; NCT03211533. Registered: July, 7th 2017. ClincalTrials.gov; NCT03234959. Registered: August, 1st 2017.

## Background

In the last decade, the progress in Information and Communication Technology (ICT) led to an increasing use of digital technologies; in particular, wearable accelerometers [1] are widely used in healthcare to quantify motion. Kinematic data obtained through wearable sensors could become crucial for detecting health status, for at least twofold reasons: (1) to support clinical assessments; (2) to continuously monitor motor parameters in ecological environments (i.e., at home) and with little invasiveness for the subject, due to their lightweight and small dimensions.

Several works exploit wearable accelerometer to monitor and assess motor behaviour, but most of them mainly concern the monitoring of toddler and pre-school children [2–4] or adults [5]; on the contrary, despite the good reliability of wearable sensors, few papers focused on their use in infant population [6].

Wearable sensors enable to study infants’ motor development during their first years of life [7]; indeed, if early signs of atypical development occur, an Early Intervention (EI) process is crucial for providing the right tailored rehabilitation program. In general, infants’ motor behavior assessment is based on clinical scales (e.g., Infant Motor Profile (IMP) or Alberta Infant Motor Scale (AIMS)), and on direct observation during playing, but quantitative and evaluator-free data are scarcely obtained. Chen et al. [8] reviewed several wearable systems which provided quantitative data in order to assess upper limb movements [9] and the ‘general movements’ [10] of infants at high risk for neurodevelopmental disorders (NDDs). These studies represent evidence of the wearable technology utility as an objective outcome measure, able to help the clinicians in the early detection of NDDs, thus allowing the start of EI. However, further investigations are required in order to increase the scientific knowledge and develop evidence-based approaches.

The EI approach is often focused on family involvement and home-based settings and it could be a useful environment to obtain quantitative measures of infant development [11]. In this context, the CareToy approach [12] represents a validated tool for personalized tele-rehabilitation and monitoring of the development of very young infants. The CareToy (CT) system was developed in the framework of a multicentric international project (www.caretoy.eu, Trial Registration: NCT01990183) and it consisted of a biomechatronic smart baby gym equipped with different types of sensors embedded in the mat, in the toys and in the gym walls. In addition, a kit of wearable sensors (i.e., two for the upper limbs and one for the trunk) was provided. The entire system was delivered to families’ houses for carrying out a customized training which was remotely monitored by the clinical and rehabilitation staff. CT has been validated in a randomized controlled trial (RCT) study [11] in which Italian and Danish preterm infants at low risk for Cerebral Palsy (CP) were involved.

**Fig. 1:**
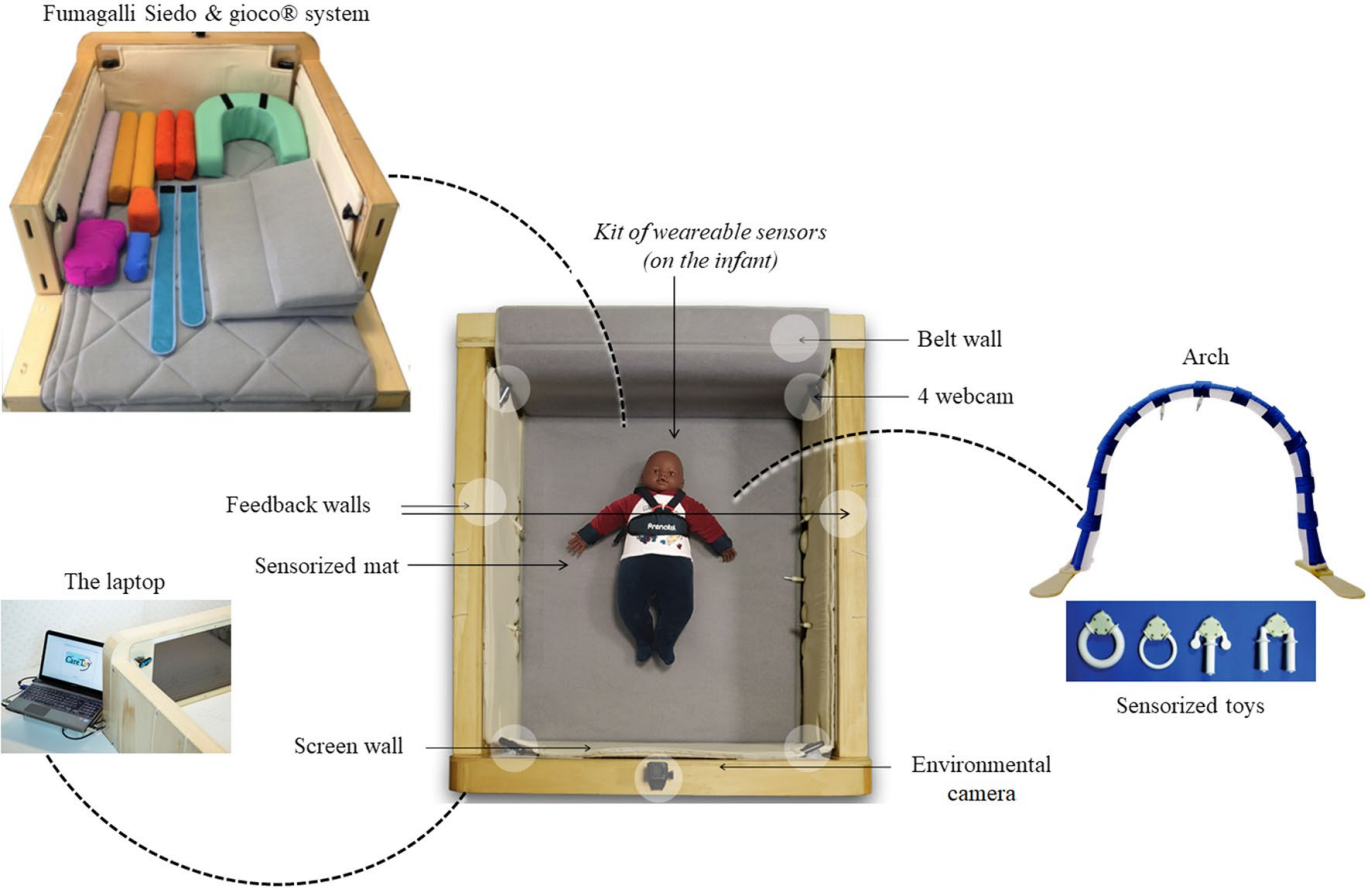
General overview of CareToy-R platform and its components

Based on previous findings, an EI program has been implemented involving infants at high-risk of CP. An adapted version of CT was proposed (Fig. 1), namely the CareToy-Revised (CT-R) version (Trial registration: NCT03211533 and NCT03234959) [13].

The activities of the CT-R training (i.e., CareToy scenarios) were highly personalized basing on the rehabilitative needs identified during the assessment and in particular on the first week of training. In details, the first period, mainly composed of the first week consisted of the use of common CT-R scenarios activities planned and carried out to explore infant’s behavior in different positions basing on the CT-R stimuli. The data of this first assessment period allowed the rehabilitation staff to better understand the infants’ needs in relation to the CT-R activities and to increasingly customize the training over the following weeks.

In the current work, we have hypothesized that the analysis of this first CT-R training period can allow to collect quantitative movement data to be compared with the clinical data. Specifically, we wanted to test that:

### H1

There is a significant relation between the accelerometric data during CT-R play and the scores of IMP and AIMS clinical assessments at baseline;

### H2

Clinical outcomes (i.e. assessment scores) can be predicted from the quantitative accelerometric data combined with baseline clinical data, such as the infants’ corrected age, prematurity and severity of brain lesion.

## Materials and methods

We specifically aimed to combine the use of accelerometers placed on the infants’ upper limbs and trunk and their temporal data sequences with the clinical assessment.

This section presents the experimental set-up and the sensors used during the training. Moreover, a description of the proposed outcome measures, the infants’ enrolment and the methods for statistical analysis are provided.

### Experimental set-up

#### Participants

Seventeen infants were included in the present study. All the subjects were recruited during the hospitalization in the Neonatal Intensive Care Units or during the neurodevelopmental follow-up programs in three University Hospitals in Tuscany (Italy): “Santa Chiara Hospital” in Pisa, the “Meyer Children’s Hospital” and the “Careggi General Hospital” in Florence. The demographic and clinical descriptions are shown in Table 1.

**Table 1:** Demographic description of subjects involved in the study with IMP and AIMS clinical assessments

Subjects	Prematurity	Sex	Pattern	Gestational age (weeks)	Corrected age at baseline in months	AIMS	IMP total	IMP var	IMP fluency	IMP symmetry
Total (n = 17)	8 preterm 9 term	12 male 5 female	6 sym 11 asym	34.78 ± 5.68 (26 ÷ 41.86)	6.35 ± 2.95 (3.12 *÷* 13.8)	11.53 ± 3.54 (7 ÷ 19)	65.69 ± 6.57 (55.34 ÷ 77.62)	64.05 ± 7.04 (53.13 ÷ 78.13)	70.10 ± 8.36 (50 ÷ 75)	80.99 ± 13.70 (47.62 ÷ 100)
Mild/ moderate brain injury (n = 7)	3 preterm 4 term	6 male 1 female	3 sym 4 asym	35.09 ± 5.43 (28 ÷ 40.71)	6.39 ± 2.44 (4.27 ÷ 10.78)	14.86 ± 2.91 (11 ÷ 19)	70.67 ± 5.33 (60.79 ÷ 77.62)	70.09 ± 5.06 (62.5 ÷ 78.13)	70.24 ± 9.45 (50 ÷ 75)	89.00 ± 8.72 (72.22 ÷ 100)
Severe brain injury (n = 10)	5 preterm 5 term	6 male 4 female	3 sym 7 asym	34.57 ± 6.13 (26 ÷ 41.86)	6.33 ± 2.82 (3.12 ÷ 13.08)	9.2 ± 1.40 (7 ÷ 11)	62.21 ± 5.01 (55.34 ÷ 70.27)	59.83 ± 4.79 (53.13 *÷* 68.75)	70.00 ± 8.05 (50 ÷ 75)	75.38 ± 14.07 (47.62 ÷ 95.24)

According to study protocol [16] the main inclusion criteria were infants with perinatal brain injury (such as cerebral hemorrhage and of Periventricular Leukomalacia (PVL), stroke, moderate/severe asphyxia) and atypical clinical signs/scores at General Movements Assessment (GMA) or Hammersmith Infant Neurological Examination (HINE). Main exclusion criteria were cerebral malformations or severe sensory impairments.

All the seventeen infants showed a brain injury on early neuroimaging, based on Ultra Sound and MRI images. On the basis of these types of neuroimaging data two different groups have been identified [17]: (1) mild/moderate brain injury, i.e. infants who presented small unilateral hemorrhagic infarction, preterm white matter injury of grade I and II [18], intraventricular hemorrhage of grade I-III [13], hypoxic-ischemic lesion and ischemic stroke but without basal ganglia engagement) and (2) severe injury, i.e. infants with preterm white matter lesion (grade III), hypoxic-ischemic injury with predominant basal ganglia-thalami pattern, expanded bilateral hemorrhagic infarction, ischemic stroke and with basal ganglia involvement or asymmetry of the internal capsule of the posterior limb.

In this study, ten subjects had severe brain injury and seven mild/moderate injury. Considering the type and location of the lesion and based on the spontaneous prevalent use of upper limb during the baseline assessments, two groups have been identified: symmetrical and asymmetrical, which included six and eleven infants, respectively, as shown in Table 1.

Before starting the CT-R training, all the infants were assessed at baseline with a standardized clinical protocol. The overall criteria for proposing the start of the training was based on motor skills, in particular at least an initial head control and no more than the mature trunk control in sitting position. The main clinical tests, reported also in the current study, were the Infant Motor Profile (IMP) and Alberta Infant Motor Scale (AIMS). The IMP is an evaluation of motor infants’ motor behaviour during different positions (supine, prone, sitting, standing while grasping and manipulating objects). It is subdivided in different motor domains and in this work we have considered the Variation, Symmetry, Performance, Fluency, and Total scores that assess movement variability and symmetry, motor abilities and motor fluency, respectively [14]. Moreover, from the IMP assessment, the AIMS—which assesses the gross-motor infant skills [15]—can be computed (Table 1).

All the parents of the enrolled infants provided a written informed consent, prior to the beginning of assessment. Ethics approval was obtained from the Tuscany Paediatric Ethics Committee, Italy (N. 84/2017).

#### CareToy R (CT-R) training

CT-R is a new system for tele-rehabilitation in young infants that makes use of highly personalized home training. In general, the training lasted 8 weeks, in which the rehabilitation staff planned the training every day for a total of 30 to 45 min per day. Every day was organized in different CT-R scenarios lasting from 2 up to 10 min each. Each scenario had a different goal and based on it, the infant could be placed in different positions inside the system: supine, prone, sitting and on one side. The family chooses the time when their infants are most collaborative and, if necessary, divides the daily training in different sessions, in order to optimize infant’s participation. As said in the background section, the first training period (generally, the first week), is essential to evaluate the infant’s adaptability within the system and to observe his/her behaviour in the enriched environment provided by the CT-R system.

#### Upper limb ICT set up

**Fig. 2:**
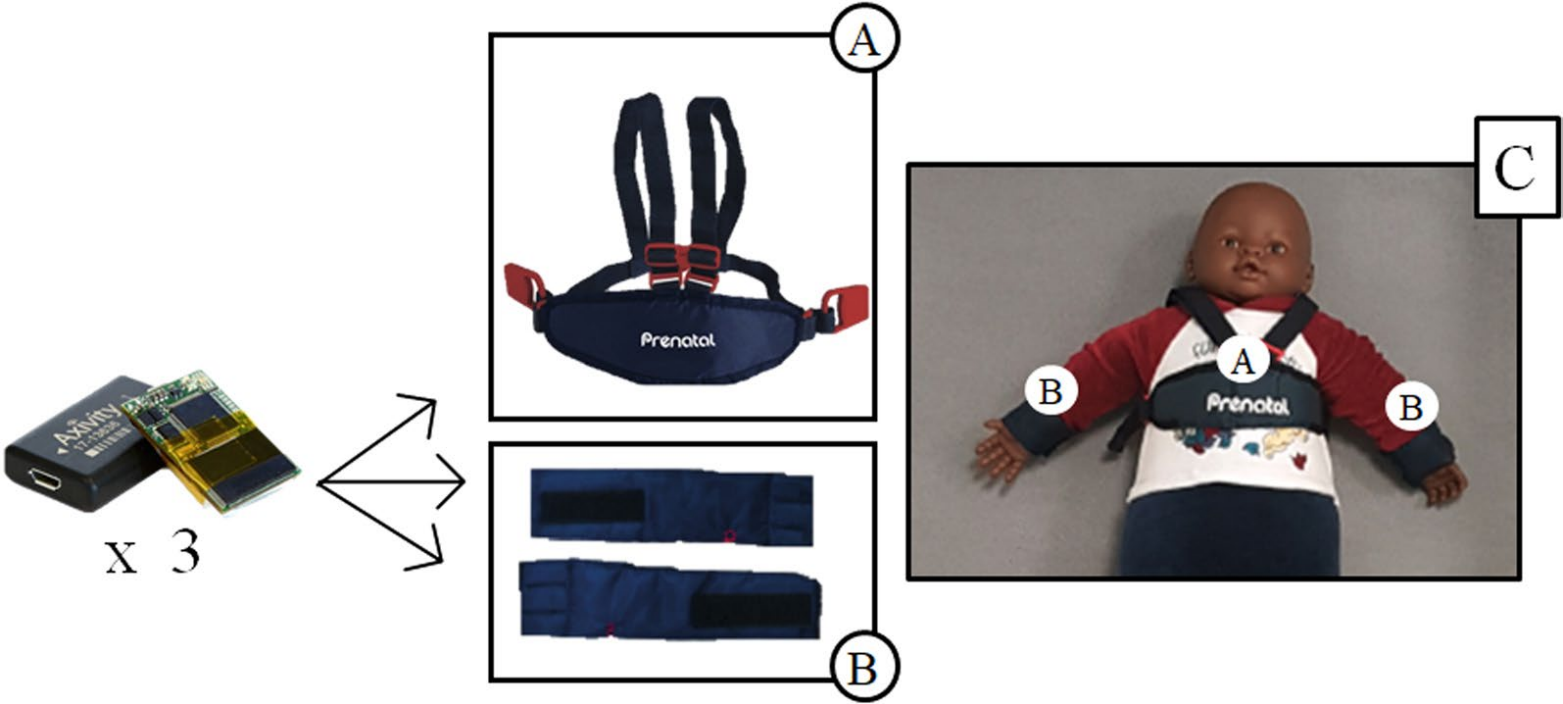
A representative example of tri-axis accelerometer placement during the study. Custom bands used as housing for the sensors applied on trunk (A) and wrists (B). Panel C shows the final acquisition setup configuration

Each participant wore a tri-axis accelerometer (AX3, Axivity, United Kingdom, UK, 35.4 × 24.2 × 8.9 mm, 11 g) on each wrist (dorsal side) and on the trunk (mid-sternum) during each training session, in order to detect the body movement (Fig. 2). Every family was provided with at least two kits, each one composed of three sensors. A commercial trunk-band (Prenatal company, Milano, IT) was customized for inserting one of the sensors (Fig. 2A) while the two other devices were applied on the body using custom-made Velcro wrist-bands (Fig. 2B). These bands were designed to be not intrusive nor to cause any sort of impairment regarding the infant’s movements. Moreover, the use of Velcro provided an excellent adherence between the sensors and the wrist in respect to a typical silicon bracelet, reducing the possibility of unwanted vibrations. The material was specifically chosen in order to eliminate any risk of an allergic reaction and to be easy to clean. The final set-up configuration is reported in Fig. 2C. Data were acquired with a sample rate of 25 Hz, in order to record continuously for at least 34 days, and sensitivity was set at +/- 8 g. This value was chosen among the possible dynamic range options provided (i.e. ±2 g, ±4 g, ±8 g, ± 16 g) as a tradeoff between sensitivity and risk of sensor saturation. The accelerometers were given to the families already fully charged and initialized; therefore, there was no need to recharge or to turn on/off the sensors before and after each training session. The first kit acquired data for around a month, then the second kit recorded the following training period.

### Data analysis

The data collected by the sensors were saved at the end of each sensor kit acquisition and visually analyzed and exported using the OmGui software (OMGUI Configuration and Analysis tool, version 43, Open Source in Microsoft.NET Framework, Microsoft Corporation, Redmond, Washington, USA). Then, they were analyzed with MATLAB software (version 9.9, The MathWorks Inc., Natick, MA, USA) by using a custom code.

We exploited the source codes provided from the Axivity website [16] to compute the Signal Magnitude Vector minus 1 g (SVM) index:

1 $$SVM=abs\left(\sqrt{{a}_{x}^{2}+{a}_{y}^{2}+{a}_{z}^{2} }-1\right)$$

where a_x_, a_y_, a_z_ are the acceleration values, expressed in units of “g” (namely, Earth standard gravitational unit), recorded along the x, y, z axes of the single tri-axis accelerometer. This index is commonly used for the analysis of daily activities.

Another measure of body movements which is widely used in healthcare is the Activity Counts (AC) (ActiGraph, Florida, FL) index, that evaluates the magnitude of body segment movements during a certain period of time called epoch, starting from acceleration data. Since AC is computed by a proprietary software, the Activity Index (AIX) was proposed and validated in [17] to provide an open source AC alternative. In this work, it was used to summarize raw tri-axial accelerometers data for each infant’s limb:

2 $$AIX\left(t;H\right)= \sqrt{max\left(\frac{1}{3}\left\{{\sum }_{m=1}^{3}\frac{{\sigma }_{m}^{2}\left(t;H\right)-{\underset{\_}{\sigma }}^{2}}{{\underset{\_}{\sigma }}^{2}}\right\},0\right)}$$

specifically, ${\sigma }_{m}^{2}\left(t;H\right)$ denotes the variance of a single participant’s acceleration signals along axis m (m = 1,2,3) in the window of length H starting at time. The length of H defines the level of resolution in the calculation of the AIX (i.e. the smallest detectable epoch) and, in this work, it was set at 1-second as in [17] to achieve a high resolution while maintaining a low computational burden.

In addition, we adopted the Asymmetry Index (AI) proposed and validated in [18] to assess quantitative evidence of an asymmetry condition in the use of two distinct body segments. By using the additivity property of the AIX, the AI computed for the i-th minute of acquisition is defined as:

3 $$AI\left(i\right)= \frac{{{sum}_{i}(AIX}_{MP})-{sum}_{i}({AIX}_{LP})}{{sum}_{i}\left({AIX}_{MP}\right)+{sum}_{i}\left({AIX}_{LP}\right)}*100$$

where ${{sum}_{i}(AIX}_{MP})$ and ${{sum}_{i}(AIX}_{LP})$ are the AIXs extracted for the more preferred (MP) and less preferred (LP) hand, respectively, summed over the i-th minute.

For each infant, a period of clinical training was defined (see CareToy R training section). Therefore, the analysis of the single infant has been restricted to that pre-calculated period.

The data of different sensors have been firstly synchronized and re-sampled at 25 Hz with a linear interpolation method; then, the AIX and SVM indexes have been computed for the first acquisition period. The SVM signals are digitally filtered with a fourth order Butterworth filter pass-band in the 0.5–10 Hz range and then are averaged per minute. The SVM was calculated to differentiate the actual training with respect to the overall period. The entire period was divided into ten-minute windows: if the SVM signal of either one of the arms exceeded an empirical threshold (set at 0.4 g to discriminize the jerky infants’ movements from possible caregiver interactions) even if only once, the entire window was considered part of the training. The isolated active windows were considered false positives; therefore, they were eliminated. An additional filter permitted to erase isolated windows of single minutes of activity. Lastly, the days of the effective training were defined as the days which contained active windows. The weeks were then merged longitudinally.

The sensor placed on the trunk has been chosen as reference, and ${AI}_{{hand}_{trunk}}$ has been determined to assess the magnitude of the single arm movement with respect to the trunk:

4 $${AI\left(i\right)}_{han{d}_{trunk}}=\frac{{{sum}_{i}(AIX}_{hand})-{sum}_{i}({AIX}_{trunk})}{{sum}_{i}\left({AIX}_{hand}\right)+{sum}_{i}\left({AIX}_{trunk}\right)}*100$$

Regarding the trunk, by defining ${AI\left(i\right)}_{{MP}_{trunk}}$ and ${AI\left(i\right)}_{{LP}_{trunk}}$ as the AI of MP and LP hand, respectively, we have estimated two more indexes to describe the total arms movement and the asymmetry between the upper limbs, namely $Total Movement\left(i\right)$ and ${AI\left(i\right)}_{{MP,LP}_{trunk}}$:

5 $$Total Movement\left(i\right)= {AI\left(i\right)}_{{MP}_{trunk}}+{AI\left(i\right)}_{{LP}_{trunk}}$$

6 $${AI\left(i\right)}_{{MP,LP}_{trunk}}= {AI\left(i\right)}_{{MP}_{trunk}}-{AI\left(i\right)}_{{LP}_{trunk}}$$

### Statistical analysis

All statistical analyses were performed in R (version 4.0.4, 2021). Functional Data Analysis [19, 20], a framework of statistical techniques dealing with data sampled from continuous curves, has been applied to demonstrate the H1 and H2 hypotheses set in Sect. 1. Specifically, functional linear models were used to describe the relation between: the (scalar) response variables, represented by the clinical assessments (i.e., AIMS total, IMP total without the adaptation domain, IMP symmetry, IMP variance, IMP fluency, IMP performance); and the predictors, (i.e.${AI}_{{MP}_{trunk}}$, ${AI}_{{LP}_{trunk}}$,${AI}_{{MP,LP}_{trunk}}$ and Total Movement) represented as curves. A single functional linear model includes one response variable and all the predictors. Predictor curves have been aligned (registered) in such a way that the beginning of each training is the same for every subject, considering the start of the training day. Only epochs where data from all subjects are available were kept, i.e., 190 time points are used for statistical modeling.

We estimated two functional linear models: (1) with functional predictors ${AI}_{{MP}_{trunk}}$, and ${AI}_{{LP}_{trunk}}$, and the scalar clinical covariates (namely assessment age, neuroimaging score and the prematurity property); (2) with functional predictors ${AI}_{MP{,LP}_{trunk}}$ and Total Movement, and the same scalar clinical covariates as before. The two models are used to investigate the quality and the amount of the association between the clinical assessment scores and the arms movements asymmetry and magnitude.

Single p-values relative to functional predictors have been obtained by computing the ANOVA table comparing the models with and without the predictor. The fit of models was measured by R^2^, and significance was set for p < 0.05.

H1 was tested by determining the significance of each functional predictors regarding each clinical outcome; H2 was examined first by defining the significance and the fit of the overall models, then by validating their predictive accuracy through leave-one-out cross validation, providing the resulting Mean Absolute Error (MAE) and Mean Absolute Percentage Error (MAPE).

## Results

**Fig. 3:**
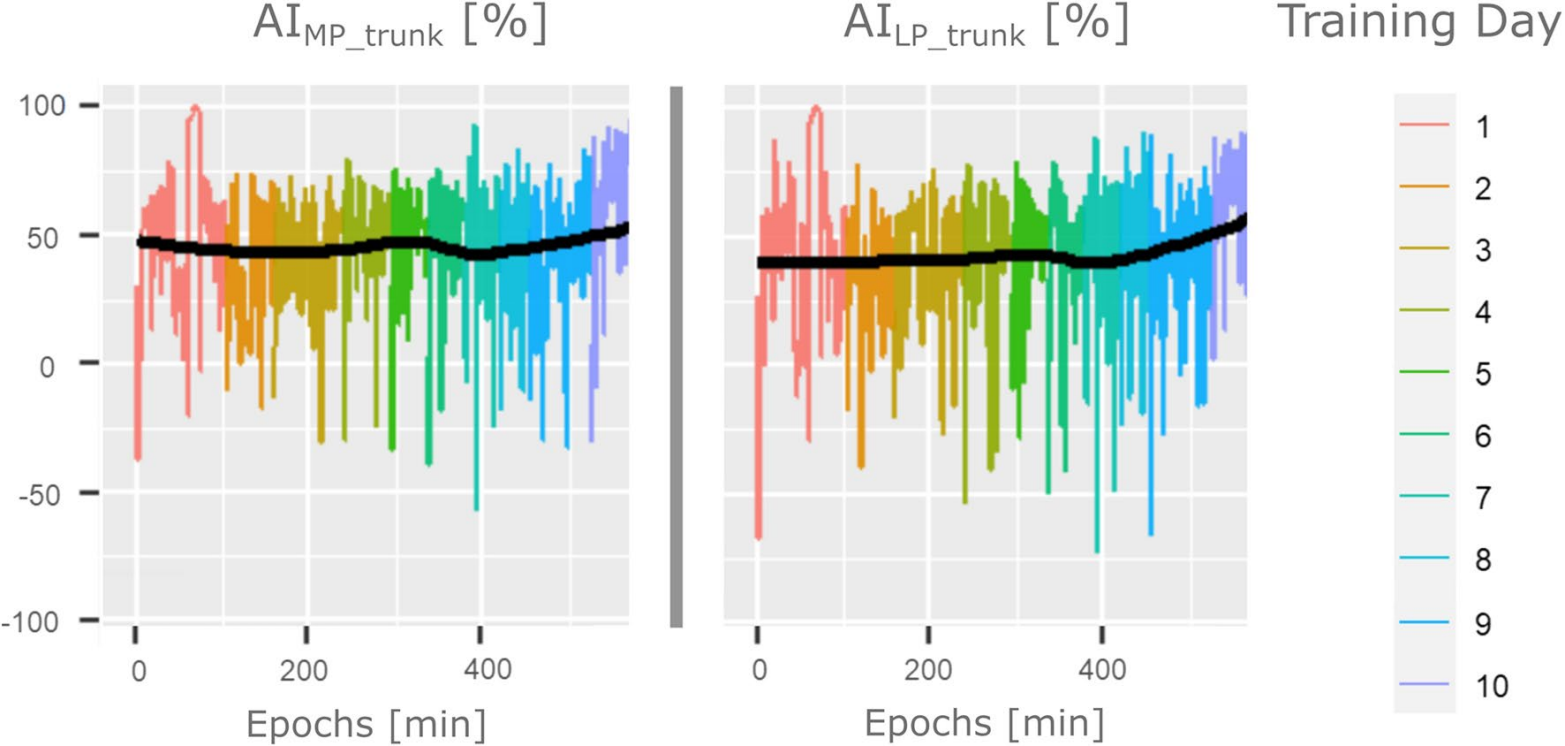
Longitudinal data of one subject’s arms: Longitudinal data of upper limbs movements in respect to the trunk of an infant born at term and classified as a class 2 severity across the first period of CT-R Training performed at the age of 38 months

Figure 3 reports the temporal trend of upper limbs activity compared to the trunk of one infant born at term, classified as class 2 severity across the first period of CT-R training performed at the age of 38 months.

**Fig. 4:**
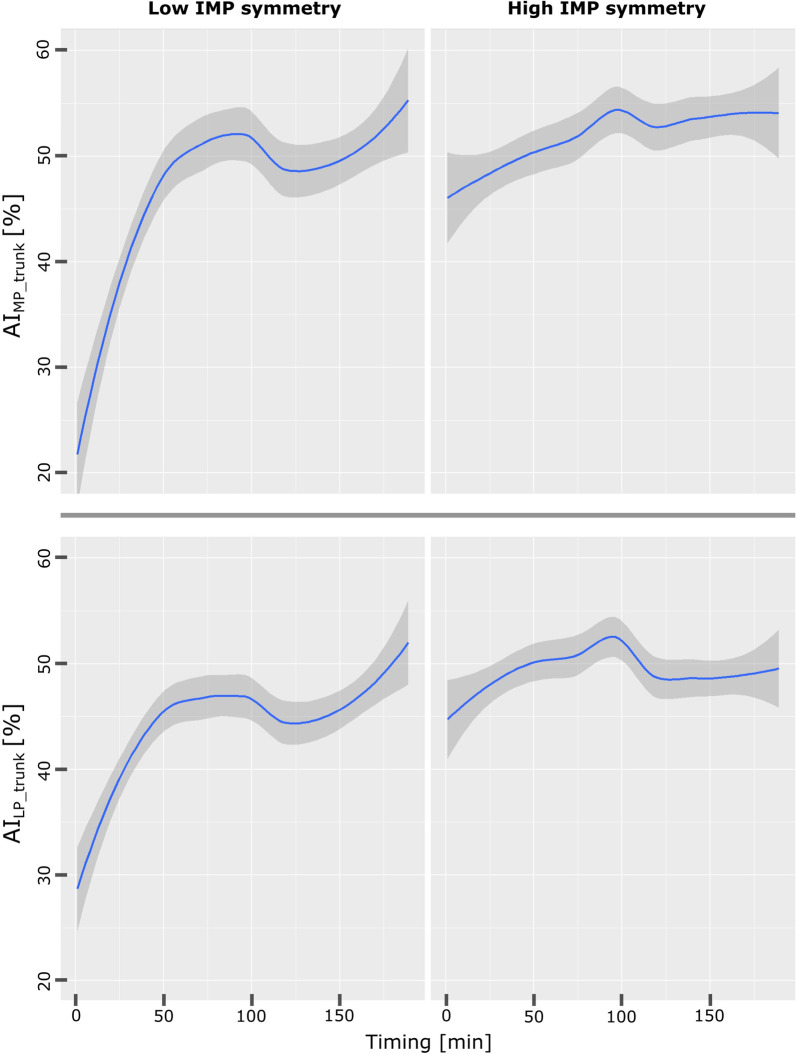
More preferred and Less Preferred hand predictors trend based on IMP symmetry scores. Moving averages (solid blue lines) and standard deviation (grey areas) of${AI}_{{MP}_{trunk}}$and ${AI}_{{LP}_{trunk}}$ predictors versus time reported for infants with high and low IMP symmetry scores

**Fig. 5:**
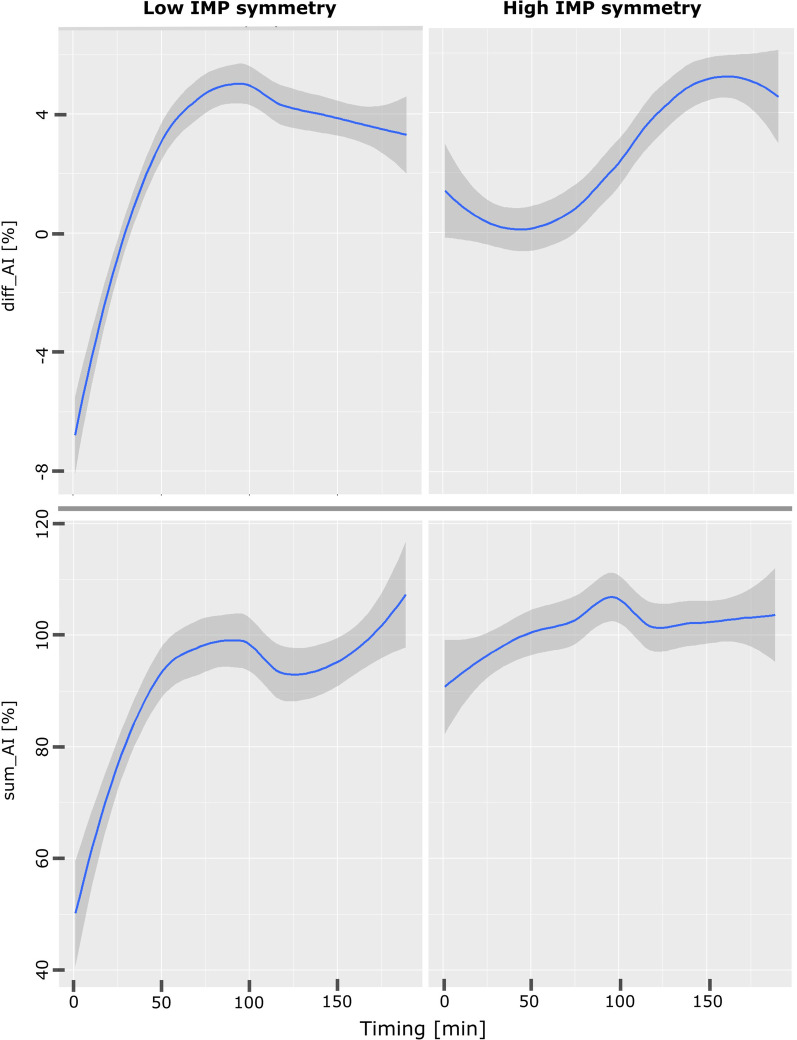
Hand asymmetry and total movement predictor trends based on IMP symmetry scores: Moving averages (solid blue lines) and standard deviation (grey areas) of ${AI}_{{MP,LP}_{trunk}}$and Total Movement versus time reported for infants with high and low IMP symmetry scores

In Figs. 4 and 5 we reported the moving averages of infants clustered by who reported low and high IMP symmetry scores as an example. It is evident in Fig. 4 how infants with inferior scores tend to use both the MP and LP arm less in respect to infants with higher IMP symmetry: the former intensify their arms usage over the period, while the latter maintain a stable upper limb usage, as confirmed by the total movement graphs in Fig. 5. Differences between the MP and LP arm use represented in Fig. 4 by the ${AI\left(i\right)}_{{MP,LP}_{trunk}}$ means are wider and unstable in the case of low scores in respect to infants which achieved higher scores in the symmetry domain.

The results provided by the statistical analysis are presented according to hypotheses H1 and H2, and then summarized in Tables 2 and 3.

**Table 2:** Resume of functional linear modeling from ${AI}_{{MP,LP}_{trunk}}$ plus Total Movement predictors combination

Model outcome	Covariate and predictors	Coefficient	p-value	Overallp-value	Adjusted R^2^	MAE	MAPE (%)
AIMS	Assessment age	− 0.02	0.827	0.001**	0.99	1.02	8.74
	Neuroimaging score	− 1.44	0.031*				
	Prematurity	− 1.77	0.019*				
	${\mathrm{AI}}_{{\mathrm{MP},\mathrm{LP}}_{\mathrm{trunk}}}$	–	0.002**				
	Total movement	–	0.009**				
IMP variability	Assessment age	− 1.01	0.169	0.034*	0.9	8.96	14.55
	Neuroimaging score	− 6.84	0.056				
	Prematurity	− 1.89	0.468				
	${\mathrm{AI}}_{{\mathrm{MP},\mathrm{LP}}_{\mathrm{trunk}}}$	–	0.143				
	Total movement	–	0.064				
IMP symmetry	Assessment age	2.38	0.139	0.044*	0.88	16.99	21.43
	Neuroimaging score	− 11.8	0.091				
	Prematurity	13.45	0.069				
	${\mathrm{AI}}_{{\mathrm{MP},\mathrm{LP}}_{\mathrm{trunk}}}$	–	0.039*				
	Total movement	–	0.154				
IMP fluency	Assessment age	2.1	0.466	0.784	− 0.5	42	62.25
	Neuroimaging score	3.48	0.754				
	Prematurity	6.95	0.546				
	${\mathrm{AI}}_{{\mathrm{MP},\mathrm{LP}}_{\mathrm{trunk}}}$	–	0.763				
	Total movement	–	0.9				
IMP performance	Assessment age	0.05	0.936	0.033*	0.9	10.6	24.74
	Neuroimaging score	2.53	0.388				
	Prematurity	− 7.26	0.064				
	${\mathrm{AI}}_{{\mathrm{MP},\mathrm{LP}}_{\mathrm{trunk}}}$	–	0.041*				
	Total movement	–	0.061				
IMP total	Assessment age	0.88	0.040*	0.004**	0.98	4.07	6.29
	Neuroimaging score	− 3.16	0.054				
	Prematurity	2.816	0.072				
	${\mathrm{AI}}_{{\mathrm{MP},\mathrm{LP}}_{\mathrm{trunk}}}$	–	0.006**				
	Total Movement	–	0.012*				

Significances are reported as *p < 0.05, **p < 0.01, ***p < 0.001

**Table 3:** Resume of functional linear modeling from ${AI}_{{MP}_{trunk}}$ plus ${AI}_{L{P}_{trunk}}$ predictors combination.

Model outcome	Covariate and predictors	Coefficient	p-value	Overallp-value	Adjusted R^2^	MAE	MAPE (%)
AIMS	Assessment age	− 0.14	0.145	0.001**	0.99	0.83	8.39
	Neuroimaging score	− 1.48	0.008**				
	Prematurity	− 2.11	0.004**				
	${AI}_{{MP}_{trunk}}$	–	<0.001***				
	${AI}_{L{P}_{trunk}}$	–	0.001**				
IMP variability	Assessment age	− 1.10	0.166	0.034*	0.9	7.36	11.86
	Neuroimaging score	− 7.03	0.039*				
	Prematurity	− 1.90	0.46				
	${AI}_{{MP}_{trunk}}$	–	0.102				
	${AI}_{L{P}_{trunk}}$	–	0.118				
IMP symmetry	Assessment age	2.85	0.191	0.044*	0.88	23.9	30.17
	Neuroimaging score	− 10.30	0.163				
	Prematurity	14.40	0.106				
	${AI}_{{MP}_{trunk}}$	–	0.068				
	${AI}_{L{P}_{trunk}}$	–	0.085				
IMP fluency	Assessment age	2.63	0.427	0.784	− 0.5	42.97	63.09
	Neuroimaging score	3.15	0.762				
	Prematurity	8.28	0.493				
	${AI}_{{MP}_{trunk}}$	–	0.763				
	${AI}_{L{P}_{trunk}}$	–	0.77				
IMP performance	Assessment age	− 0.28	0.598	0.033*	0.9	7.2	16.82
	Neuroimaging score	2.23	0.251				
	Prematurity	− 8.17	0.019*				
	${AI}_{{MP}_{trunk}}$	–	0.015*				
	${AI}_{L{P}_{trunk}}$	–	0.013*				
IMP total	Assessment age	1.02	0.030*	0.004**	0.98	3.45	5.37
	Neuroimaging score	− 2.99	0.041*				
	Prematurity	3.15	0.049*				
	${AI}_{{MP}_{trunk}}$	–	0.004**				
	${AI}_{L{P}_{trunk}}$	–	0.004**				

Significances are reported as *p < 0.05, **p < 0.01, ***p < 0.001

Regarding H1, scores of clinical assessments at baseline showing significant relation with accelerometric data were mainly AIMS total and IMP symmetry, performance and total. Such relations were, however, different in quality and quantity. AIMS total was significantly with all the functional predictors (p < 0.01). IMP symmetry is significantly associated with ${AI}_{{MP,LP}_{trunk}} (p<0.05)$, while the domain IMP performance was correlated significantly to ${AI}_{{MP,LP}_{trunk}}$ and to both ${AI}_{{MP}_{trunk}}$and ${AI}_{{LP}_{trunk}} (p<0.05)$. The IMP total showed a solid connection to all the accelerations variables, reaching higher significances for ${AI}_{{MP,LP}_{trunk}}$, ${AI}_{{MP}_{trunk}}$and ${AI}_{{LP}_{trunk}} (p<0.01)$. The IMP variability and IMP fluency have not shown any relation to the accelerometers data.

Focusing on H2, except for the IMP fluency domain, all the overall models were significant in determining the clinical scale outcomes. Both AIMS total models were highly significant, especially in case of ${AI}_{{MP}_{trunk}}$and ${AI}_{{LP}_{trunk}}$as predictors (Adjusted R^2^ = 0.99, F(13,3) = 234.9, p < 0.001) with an evident correlation to the neuroimaging score and the prematurity property (p < 0.01) clinical data. The models were significant even in case of IMP variability outcome, primarily due to the neuroimaging score (p < 0.05) especially in case of ${AI}_{{MP}_{trunk}}$and ${AI}_{{LP}_{trunk}}$predictors (Adjusted R^2^ = 0.91, F(13,3) = 13.09, p < 0.05). On the contrary, the IMP symmetry model was significant thanks to the ${AI}_{{MP,LP}_{trunk}}$ influence (Adjusted R^2^ = 0.91, F(13,3) = 8.38, p < 0.05). Both the IMP performance models were considered valid, with higher fit and significance showed in case of using the ${AI}_{{MP}_{trunk}}$and ${AI}_{{LP}_{trunk}}$predictors combination (Adjusted R^2^ = 0.95, F(13,3) = 11.66, p < 0.05) with a significant contribution given by the prematurity information (p < 0.05). Lastly, IMP total models were highly significant for both the ${AI}_{{MP,LP}_{trunk}}$ and Total Movement (Adjusted R^2^ = 0.98, F(13,3) = 49.71, p < 0.01) and ${AI}_{{MP}_{trunk}}$and ${AI}_{{LP}_{trunk}}$predictors (Adjusted R^2^ = 0.98, F(13,3) = 60.97, p < 0.01). In particular, all the clinical data concurred significantly in the determination of this last model. Neither one of the IMP fluency models showed any significance.

The models with AIMS and IMP total as outcomes reached a low mean error validation for both the predictors combinations (MAPE < 10%). The error associated with IMP variability was lower in case of ${AI}_{{MP}_{trunk}}$ plus ${AI}_{{LP}_{trunk}}$ predictor combination (MAPE < 15%), while IMP symmetry and IMP fluency showed lower accuracies.

## Discussion

In the last years, few works aimed at detecting non-typical motor development in infants in an automatic way, based on accelerometric features in accordance with the results obtained with validated clinical tools [21–23]. Airaksinen et al. [24] trained a deep learning algorithm to determine a novel scale, the Baba Infant Motor Score (BIMS), starting from the data obtained by the MAIJU wearable system; the predicted BIMS scores showed a significant linear correlation with the AIMS scores (r = 0.83, p < 0.01). To our knowledge, this is one of the first works that aim to find a correlation between the AIMS and the IMP clinical scales with accelerometric data (H1) and to create a model that can predict granularly these clinical assessments by implementing objective data (H2).

As shown in the results section, the prediction of the AIMS outcome (i.e., a general measurement of infants’ gross-motor abilities), is statistically significant while considering the arms difference movement and their total movement as predictors, respectively. The most significant model that predicts the AIMS outcome is generated by combining the MP hand movements with the LP ones. In this model, even the contribution of the brain injury severity and prematurity is significant. As clinically expected, the correlation between the AIMS and the severity of brain injury is negative for both the presented models, asserting that infants with more severe damage are likely to have lower AIMS scores compared to the ones with no lesion. However, the models also state that the correlation between the outcome and the term/preterm infants is negative, thus preterm infants should have higher AIMS scores with respect to infants born at full-term. This statement is in contrast with the clinical experience, but these results could be influenced by the fact that all full-term infants enrolled in this study report more severe brain damage compared to the preterm ones. Lastly, the assessment age covariate is not significant for none of the models.

Regarding the IMP scale as outcome (i.e., evaluation of spontaneous motor behaviour), we analyzed each of its domains (i.e., variation, symmetry, performance, fluency, and total scores). Overall, our findings show statistical significance within almost all domains except for the fluency one. Indeed, the models which consider the IMP fluency denote a lack of correspondence between the infant’s motor fluency and the indexes based on the arms accelerations; conversely, the IMP variation domain presents total models statistically significant for both the predictors combination, but it is only the brain injury severity covariate that has a significant impact in the determination of the outcome for both the models. These results suggest that the IMP variation scores are strongly connected with only the infant’s brain lesion severity and not with the temporal trend of the acceleration data.

Instead, concerning IMP symmetry, performance and IMP total score (without the adaptability domain) as outcome, we can notice correlations of the global model when the movement difference is combined with the total movement. For all three outcomes, arms movement difference is determinant to the significance of the model with respect to the total movement predictor and the clinical covariates. However, the IMP performance model shows further significance when single upper limb movements were considered along with the term-preterm covariance, whose coefficient shows a negative correlation. IMP fluency, however, presents a negative fit that implies a strong discordance between both the single arm movement magnitudes and their combination.

Statistical models also showed good accuracy in case of outcome AIMS e IMP total, providing clear indication for robust predictive performance.

Based on all these findings, we demonstrated that:

### H1

There is a significant correlation between the accelerometric data during CT-R play and the clinical assessment scores at baseline. This is particularly evident for the AIMS and the IMP total, as given by the general statistical significance assessed by the predictors p-values;

### H2

It can be generated a model that permits to estimate the outcome (i.e. clinical assessment scores) automatically from the quantitative accelerometric data combined with baseline clinical data, such as the infants’ corrected age, prematurity and severity of brain lesion. This is true for all the considered clinical scales, apart from the IMP Fluency domain, as confirmed by the overall significance of p-values. The validation results state that for the AIMS and the IMP total clinical scales the models could already be assessed by the presented models. On the contrary, for the other scores and, in particular, for the IMP Fluency domain, more subjects are necessary in order to provide a robust model that can be used in the clinical reality.

In addition, we are able to draw the following considerations: (1) a general significant association between the acceleration data and the investigated clinical assessments occurs, confirming that wearable sensors are able to recognize infants’ motor impairments, quantified by the clinical scales; (2) our approach is able to discern an asymmetry (given by the difference between the arms movement) by correlating the sensors’ data with a validated clinical assessment, suggesting that is possible to rely solely on the sensor data with respect to the studied clinical evaluations (in particular, the brain damage severity); (3) the negative correlation between brain injury and model outcome means that higher clinical scores correspond to a minor severity, as confirmed by the clinical experience, while the negative correlation between prematurity and model outcome is in contrast with the clinical experience, as reported in [25]; (4) according to [26] the weight curves have an interesting significance because they could help to determine certain periods that are more influential in the determination of the model outcome.

Finally, the resulting models provided in this paper can be considered one of the first steps for providing diagnostic decision support tools in the infant rehabilitation framework that can aid the clinician staff in determining upper limbs motor abilities with more accuracy basing on the objective data coming from the accelerometers. In a tele-rehabilitation approach, the models proposed could allow the constant monitoring of the child’s motor abilities while he\she plays across the entire training period.

Although the results collected so far are encouraging, we are aware of some relevant limitations that still prevent a general applicability of this work. Firstly, we had too few data to claim a full validation of the presented models and there is also a high variability in the clinical assessments among infants. Moreover, there was not a control group to make a comparison between the typical and atypical developmental infants’ behavior. Lastly, these data were recorded while the infants were performing different kinds of goal-directed activities, not temporally correlated among them. In other words, in a certain minute, two different infants were likely performing two different activities. This is coming as a direct consequence of the foundation of the CT-R idea, which is to provide each infant with the best personal and tailor-suited playtime activities for giving them their appropriate developmental needs. Thus, this intrinsic limitation may be also considered the great power of the proposed model: no matter what game is played or what movement is executed, the use of the simple accelerometers could still predict a valid outcome.

## Conclusions

The main goal of our work was to propose a novel technique to combine the use of acceleration sensors and a statistical analysis method by exploring the relation between temporal data sequences and the clinical assessment. To date, no works proposed the use of simple accelerometers for studying infants’ motor assessment and development in an ecological unstructured environment, such as the CT-R. In this framework, the infants, wearing non-invasive tri-axial accelerometers on each wrist and on the trunk, were able to play without constraints while the sensors recorded their upper limbs and trunk movements without any external help.

Future works will focus on the enrolment of a higher number of infants to create a robust dataset in order to fully validate the proposed data analysis and determine the clinical assessment score in an ecological unstructured environment (i.e. at home). Moreover, from a tele-rehabilitation perspective, a new device could be developed to save and transfer data wirelessly to a secure server for the clinicians and the rehabilitative staff use. In this way, the monitoring of the infants’ motor behavior would be constant and easy to check every time and in every place by means of a portable device, like a smartphone.

## Data Availability

The datasets used and/or analysed during the current study are available from the corresponding author on reasonable request.

## Abbreviations

AC: Activity Counts
AIMS: Alberta Infant Motor Scale
AI: Asymmetry Index
AI_MP,trunk_: Asymmetry Index of more preferred hand respect to the trunk
AI_LP,trunk_: Asymmetry Index of less preferred hand respect to the trunk
AIX: Activity Index
AIX_MP_: Activity Index more preferred hand
AIX_LP_: Activity Index less preferred hand
AIX_hand_: Activity Index of the single limb
BIMS: Baba Infant Motor Score
CP: Cerebral Palsy
CT: CareToy
CT-R: CareToy-Revised
EI: Early Intervention
GMA: General Movement Assessment
HINE: Hammersmith Infant Neurological Examination
ICT: Information and Communication Technology
IMP: Infant Motor Profile
IMU: Inertial Measurement Unit
LP: Less Preferred
MAE: Mean Absolute Error
MAPE: Mean Absolute Percentage Error
MP: More Preferred
NDDs: Neurodevelopmental disorders
PVL: Periventricular Leukomalacia
RCT: Randomised Controlled Trial
SVM: Signal Magnitude Vector minus 1 g

## References

[1] Rast FM, Labruyère R (2020). Systematic review on the application of wearable inertial sensors to quantify everyday life motor activity in people with mobility impairments. J Neuroeng Rehabil.

[2] Trost SG, Fees BS, Haar SJ, Murray AD, Crowe LK (2012). Identification and validity of accelerometer cut-points for toddlers. Obesity.

[3] Braito I, Maselli M, Sgandurra G, Inguaggiato E, Beani E, Cecchi F (2018). Assessment of upper limb use in children with typical development and neurodevelopmental disorders by inertial sensors: a systematic review. J Neuroeng Rehabil.

[4] Trost SG, Zheng Y, Wong W-K (2014). Machine learning for activity recognition: hip versus wrist data. Physiol Meas.

[5] Johansson D, Malmgren K, Alt Murphy M (2018). Wearable sensors for clinical applications in epilepsy, Parkinson’s disease, and stroke: a mixed-methods systematic review. J Neurol.

[6] Redd CB, Karunanithi M, Boyd RN, Barber LA (2021). Technology-assisted quantification of movement to predict infants at high risk of motor disability: a systematic review. Res Dev Disabil.

[7] Allievi AG, Arichi T, Gordon AL, Burdet E (2014). Technology-aided assessment of sensorimotor function in early infancy. Front Neurol..

[8] Chen H, Xue M, Mei Z, Bambang Oetomo S, Chen W (2016). A review of Wearable Sensor Systems for Monitoring Body movements of neonates. Sensors.

[9] Rihar A, Sgandurra G, Beani E, Cecchi F, Pašič J, Cioni G (2016). CareToy: Stimulation and Assessment of Preterm Infant’s activity using a Novel Sensorized System. Ann Biomed Eng.

[10] Gao Y, Long Y, Guan Y, Basu A, Baggaley J, Ploetz T (2019). Towards Reliable, Automated General Movement Assessment for Perinatal Stroke Screening in Infants using Wearable Accelerometers. Proc ACM Interact Mob Wearable Ubiquitous Technol.

[11] Sgandurra G, Lorentzen J, Inguaggiato E, Bartalena L, Beani E, Cecchi F (2017). A randomized clinical trial in preterm infants on the effects of a home-based early intervention with the “CareToy System” van Wouwe JP, editor. PLoS ONE..

[12] Cecchi F, Sgandurra G, Mihelj M, Mici L, Zhang J, Munih M (2016). CareToy: an Intelligent Baby Gym: home-based intervention for infants at risk for Neurodevelopmental Disorders. IEEE Robot Automat Mag.

[13] Sgandurra G, Beani E, Giampietri M, Rizzi R, Cioni G, the CareToy-R Consortium (2018). Early intervention at home in infants with congenital brain lesion with CareToy revised: a RCT protocol. BMC Pediatr.

[14] Heineman KR, Bos AF, Hadders-Algra M (2008). The Infant Motor Profile: a standardized and qualitative method to assess motor behaviour in infancy. Dev Med Child Neurol.

[15] Piper MC, Pinnell LE, Darrah J, Maguire T, Byrne PJ (1992). Construction and validation of the Alberta Infant Motor Scale (AIMS). Can J Public Health.

[16] Axivity | Product Downloads [Internet]. https://axivity.com/downloads/ax3. Accessed 29 Jul 2022.

[17] Bai J, Di C, Xiao L, Evenson KR, LaCroix AZ, Crainiceanu CM (2016). An activity index for raw accelerometry data and its comparison with other activity metrics. PLoS ONE.

[18] Beani E, Maselli M, Sicola E, Perazza S, Cecchi F, Dario P (2019). Actigraph assessment for measuring upper limb activity in unilateral cerebral palsy. J Neuroeng Rehabil.

[19] Ramsay JO, Silverman BW (2005). Functional data analysis.

[20] Ramsay JO, Hooker G, Graves S (2009). Functional data analysis with R and MATLAB.

[21] Singh M, Patterson DJ. Involuntary gesture recognition for predicting cerebral palsy in high-risk infants. International Symposium on Wearable Computers (ISWC) 2010. Seoul, Korea (South): IEEE; 2010, p. 1–8. http://ieeexplore.ieee.org/document/5665873/. Accessed 29 Jul 2022.

[22] Heinze F, Hesels K, Breitbach-Faller N, Schmitz-Rode T, Disselhorst-Klug C (2010). Movement analysis by accelerometry of newborns and infants for the early detection of movement disorders due to infantile cerebral palsy. Med Biol Eng Comput.

[23] Gravem D, Singh M, Chen C, Rich J, Vaughan J, Goldberg K (2012). Assessment of infant movement with a compact wireless accelerometer system. J Med Devices.

[24] Airaksinen M, Gallen A, Kivi A, Vijayakrishnan P, Häyrinen T, Ilén E (2022). Intelligent wearable allows out-of-the-lab tracking of developing motor abilities in infants. Commun Med.

[25] Fitzgerald E, Boardman JP, Drake AJ (2018). Preterm birth and the risk of neurodevelopmental disorders—-is there a role for epigenetic dysregulation?. CG.

[26] Dziak JJ, Coffman DL, Reimherr M, Petrovich J, Li R, Shiffman S (2019). Scalar-on-function regression for predicting distal outcomes from intensively gathered longitudinal data: Interpretability for applied scientists. Statist Surv..

